# Job satisfaction of village doctors in a rural-oriented tuition-waived medical education program in China

**DOI:** 10.3389/fpsyg.2023.1184430

**Published:** 2023-07-25

**Authors:** Renmin Jin, Yan Chen

**Affiliations:** ^1^Party School of Anhui Provincial Committee of C.P.C, Hefei, China; ^2^Anhui Medical College, Hefei, China

**Keywords:** village doctors, job satisfaction, rural-oriented tuition-waived medical education, China, health equity

## Abstract

**Introduction:**

To address health inequity and relieve shortage of rural doctors, China initiated Rural-oriented Tuition-waived Medical Education (RTME) to train doctors in rural areas for free since 2010. Little is known about job satisfaction of this particular group of rural doctors.

**Methods:**

Job Satisfaction Questionnaires for Village Doctors were distributed to 240 village doctors with RTME program in China, and 40 received in-depth semi-structured interviews. Descriptive analysis, chi-square test, univariate and multivariate logistic regressions in SPSS23.0 were conducted, and thematic analysis was applied to interviews.

**Results:**

Job satisfaction rate of village doctors with RTME program was 56.50%. Full understanding of RTME policy, recognition of rural medical work, relevance of RTME curriculum with present job, education background, rural origin and renumeration were positively correlated with job satisfaction. Preferential policies of RTME program, relaxing working atmosphere, more promotion opportunity, and easier access to higher technical titles were the key factors to retain rural doctors.

**Conclusion:**

Our findings may interest healthcare authorities, medical colleges and primary healthcare establishments. Studying the job satisfaction of village doctors in China may also be beneficial in developing community and rural health services, and provide valuable insights into the training and retention of primary healthcare providers in other countries.

## Introduction

Health equity has increasingly become one of the prominent objectives in health care reform in the world (Ke, 2008). Shortage of rural doctors is a worldwide issue in health care delivery (WHO, 2009). According to World Bank statistics, around 44% of people in the world currently live in rural and remote areas as of 2020, but most health workers live and work in cities, which has further caused health inequity.

As of 2021, China was the largest agricultural country in the world with 509.79 million rural residents, which accounted for 36.11% of the total population. It is essential to improve rural health services in China to deal with critical challenges. “*Outline of the Healthy China 2030 Plan*” promotes equalization of basic public health focusing on rural areas.

The health workforce is the social and technical foundation of any health system (Kabene et al., 2006), and the availability of sufficient human resources for the delivery of health services is a major global policy concern (Frenk et al., 2003). Increasing human resources has been identified as a high-priority area for health systems in many countries (Regan et al., 2010). Difficulties of both recruitment and retention of physicians in rural areas are well acknowledged by countries all over the world (WHO, 2009). Inadequacy of health workers with a suitable skill set in rural hospitals has been a major impediment to the implementation and achievement of the policy goals of China’s healthcare reform (Yip et al., 2012; Xu et al., 2013; Zheng et al., 2015). Village doctors are general practitioners in the rural areas and serve as the bottom-tier of the Chinese rural health system, whose main duty is to provide public health services, general disease diagnosis and treatment to rural residents. Village doctors are the indispensable health personnel for the vast majority of rural residents in China.

China has taken a series of measures to train and retain village doctors. The *Rural-oriented Tuition-waived Medical Education* (*RTME*) program is one of them, which was initiated in 2010 to enroll students to study in designated medical schools for 3 or 4 years to pursue a diploma or a bachelor’s degree and then work in village hospitals for 6 years after graduation. In this programme, the medical students would sign a contract with the medical training college and local health administrative department. The government would waive the related tuition fee for the students and provide them with a certain amount of living allowance during their studies in designated medical schools. The RTME mainly recruits students with rural backgrounds who have reached the minimum passing score in college entrance exams and subsidizes their tuition to finish a 4-year medical undergraduate education or 3-year medical diploma education in colleges (basically 1 year theory + 2-year internship). In return, these RTME students must attend a 3-year or 2-year residency in general practice (GP), which is counted into the service period, and compulsorily serve in rural township health centers for a period of 3 to 4 years; that is 6 years in total. They can apply for township assistant practicing physician license 1 year after graduation and do real job as a physician.

Despite various efforts, according to *China Health Statistical Yearbook 2021*, the number of village doctors in China has been on the decline since 2011, and there was a decrease of 205,000 village doctors in 2020 in comparison with that of 2016. *Research Report of Living Conditions of Village Doctors*, compiled by the Academy of Sciences of China in 2021, reported that men accounted for 70.8, and 96.04% village doctors expressed dissatisfaction towards the job. The fourth National Health Services Survey in China revealed that 12.2% village doctors had turnover intention, higher than that of urban doctors.

Job satisfaction is defined as a personal positive subjective evaluation or attitude towards all aspects of a work environment (Hoppock, 1935). Assessment of physicians’ job satisfaction is one of the approaches to look into existing healthcare situations and possible problems (Goetz et al., 2016). Higher satisfaction helps stabilize the medical service team, improve the satisfaction of doctors and patients, so as to ensure the quality and efficiency of medical services, and promote the implementation of medical reform policies (Zhang and Fang, 2016). Lower job satisfaction will jeopardize work motivation, and may even impact primary health work negatively as a whole (Meng et al., 2009 Gili et al., 2016). Many empirical studies on medical staff have identified that job satisfaction is negatively related to turnover intention (Mobley, 1977; Xu et al., 2013; Chen et al., 2015; Gualano et al., 2016; Zhang and Fang, 2016; Han et al., 2018 Ding et al., 2021). Village doctors report higher job satisfaction and retention rate (Chen et al., 2015; Zhang and Fang, 2016; Zhang, 2018 Gu et al., 2019).

Similar to Minnesota-Duluth Program in America, Jichi Program in Japan and CPIRD Program in Thailand, the RTME program has contributed substantially in training qualified general practitioners in rural areas in China, filled the gap of understaffed village hospitals and built a high-quality team of village doctors. However, studies on the RTME program are rather scant, mostly focusing on on-campus medical students with the RTME program in terms of curriculum design (Huang et al., 2015; Xiong et al., 2016; Hu et al., 2018; Qin et al., 2020), professional identity (Tang et al., 2019; Huai et al., 2020), and employment intention (Wang and Wen, 2016 Zhang and Wang, 2020).

It is of great significance to probe into the job satisfaction and its influencing factors of village doctors, and take corresponding measures to retain village doctors and achieve health equity. This study, by a mixed quantitative and qualitative method, focused on rural doctors in the RTME program in China, a national rural-oriented physician education policy to train primary care physicians free of charge to address the shortage of rural doctors, involving a 3-year study (basically 1-year theory + 2-year internship) in medical college, and 6-year service (2-year residency + 4-year general practitioner) in village hospitals. The participants invited in this study are those who have obtained township assistant practicing physician license.

It aims to investigate the job satisfaction of rural doctors in the RTME program, and explore the influencing factors of job satisfaction of this particular group, thereby serving as reference for relevant authorities to optimize policies and provide individualized training, in order to maintain the stability of primary healthcare staff. It may also be beneficial in developing community and rural health services, and providing valuable insights into the training and retention of primary healthcare providers in other countries.

## Methods

### Design and participants

A quantitative and qualitative design was used for this study. Stratified random sampling was used for quantitative study. Six cities in Anhui, China were selected, 3 counties in each city were selected, 3 towns in each county were selected, and at least 2 male village doctors were selected from each town. Altogether 240 village doctors graduating from the RTME program with diploma in medical science and working in village hospitals after graduation with township assistant practicing physician license, were chosen to fill out the questionnaires. Participant recruitment was carried out via WeChat, a social media platform in China, with a brief description of the study, necessary disclosures, and a direct link to the questionnaire in Wenjuanxing, an online survey application, after receiving approval from the health system’s review boards.

Semi-structured interviews were conducted online in order to get a better idea of the interviewees’ points of view about the questions in the questionnaire. To make sure the sub-sample statistics were not biased, we first randomly chose 4 interviewees from each cities, this made a sub-sample of 24 (4*6) interviewees. Then we conducted a *t*-test between the sub-sample and the full sample, and identified that there were modest significant differences in such variables as salary and educational background. Therefore, more participants were randomly added until there was no significant difference in main explanatory variables between these two samples and finally made a sub-sample of 40 interviewees.

Approval for this study was obtained from local health commissions (Decision no: 2021. 09. 30). Written informed consents were obtained from all the participants.

### Instruments

Job Satisfaction Questionnaires for Village Doctors, a well-received measurement tool that has been used in prior studies in China, was used in this study. It was developed by Doctor He Zhang in 2020 with satisfactory reliability and validity (Cronbach *α* = 0.922; *p* < 0.05; KMO = 0.89).

The questionnaire includes three sections. First, basic information, covering age, marital status, place of origin (rural or urban), educational background, length of service, etc. Second, comments on the RTME program and present job, including good understanding of relevant policies when enrolling in RTME, recognition of rural medical work, reasons for attending RTME, acceptance of required years of service in village hospitals, relevance of training provided in RTME with present job, willingness to recommend others to attend RTME, salary, main problems at work and future development. Third, job satisfaction, whose items are ranked on a 5-point Likert scale of which 1 is negatively stated (with 1 = strongly disagree to 5 = strongly agree). The higher the score, the more positive the job satisfaction.

Three research group members with teaching experience or medical background did semi-structured interviews with 40 interviewees with an outline listed as follows:

1. Why did you attend the RTME program?
2. Will you recommend the RTME program to others? Why or why not?
3. What do you think of the curriculum offered in the RTME program?
4. What are the benefits of working in village hospitals?
5. What are the main problems or difficulties of working in village hospitals?
6. Will you continue working in the village hospitals when the contract expires? Why or why not?
7. What suggestions do you have for the RTME program or to the medical students with this program?

### Data collection

The data were collected from October 2021 to April 2022. Prior to data collection, all the participants were provided with informed consent and reminded of the right to withdraw during data collection. The link of the questionnaire was sent to all the participants via WeChat, a social media platform in China. Of the 240 village doctors in the RTME program invited to participate, 223 responded to the survey properly. Questionnaire-reclaiming efficiency was 92.91 percent.

The final 40 interviewees were then invited to participate in a 30 min semi-structured interview one by one through Tencent Meeting, a popular social networking tool in China. All the interviews were recorded upon the interviewees’ approval.

### Data analysis

SPSS23.0 was used for data entry and data analysis. Statistical methods include descriptive analysis by means of constituent ratio, mean value and standard deviation; chi-square test to screen influencing factors; univariate logistic regression analysis into influencing factors; and multivariate logistic regression to identify independent influencing factors as a robust check. The qualitative data of semi-structured interviews were analyzed using thematic analysis: data transcription, initial coding, theme search, theme review and theme defining.

## Results

### Quantitative findings

#### Demographic characteristics

Demographic characteristics of the respondents, 223 in total, are as follows: 189 (84.75%) village doctors in the RTME program have siblings; 207 (92.83%) are from rural areas; 139 (62.33%) have junior college diplomas; 162 (72.65%) doctors have worked in village hospitals for 2 to 4 years (Table 1).

**Table 1:** Socio-demographic characteristics of village doctors (*N* = 223).

Variables	Category	Number	Percentage %
Age	35 and above	13	5.83
	30–34	18	8.07
	25–29	184	82.51
	24 and below	8	3.59
Place of Origin	Rural	207	92.83
	Urban	16	7.17
Educational Background	3 year Diploma	139	62.33
	Bachelor degree	84	37.67
Whether they are the Only Child	Yes	34	15.25
	No	189	84.75
Marital Status	Single	110	49.33
	Married	113	50.67
Years of service	<1	28	12.56
	2–4	162	72.65
	>5	33	14.79

#### Problems and demands

The prominent problems at work were short age of medicine and equipment (161 out of 223, 72.19%), and inadequate number (124 out of 223, 55.61%) and quality (151 out of 223, 67.71%) of medical staff. Doctors who clearly stated that they would stay in primary healthcare establishments when the contract expired only accounted for 31.84% (71 out of 223). Other preferred future career paths included seeking a position in large-scale hospitals (53 out of 223, 23.77%), pursuing a master’s degree (29 out of 223, 13.00%), and becoming a civil servant or a teacher (18 out of 223, 8.07%) (Table 2).

**Table 2:** Problems and demands of village doctors (*N* = 223).

Variables	Category	Number	Percentage %
Problems at work	No problems	2	0.89
	Patients did not follow orders	74	33.18
	Shortage of medicine and equipment	161	72.19
	Understaffed medi-care team	124	55.61
	Inadequate skill of medical staff	151	67.71
	Others	31	13.90
Desired preferential policies	Improve salary and benefits	192	86.09
	Provide housing	127	56.95
	Offer opportunity for promotion	117	52.47
	Provide training chances	171	76.68
	Pursue further studies	134	60.09
	Others	16	7.16
Plan after the contract expires	Stay in primary health care establishments	71	31.84
	Seek position in large-scale hospitals	53	23.77
	Become civil servants or teachers	18	8.07
	Pursue master’s degree	29	13.00
	Others	52	23.32
Prospects of primary health care establishments	Very pessimistic	29	13.00
	Pessimistic	45	20.18
	Neutral	64	28.70
	Optimistic	49	21.97
	Very optimistic	36	16.15

#### Job satisfaction

The total score of job satisfaction of 223 village doctors in the RTME program was (22.28 ± 8.59). The top three variables were the job itself (2.64 ± 1.03), relationship with colleagues (2.41 ± 0.76) and work-related pressure (2.31 ± 1.01). Variables with the lowest score were salary and benefits (1.82 ± 0.83), social status (2.04 ± 0.87), and long-term engagement in primary healthcare (2.12 ± 0.89) (Table 3). Job satisfaction rate of village doctors in the RTME program was 56.50% (126 out of 223).

**Table 3:** Descriptive analysis of job satisfaction of village doctors (*N* = 223).

Variables	Mean ± SD
The job itself	2.64 ± 1.03
Relationship with colleagues	2.41 ± 0.76
Work-related pressure	2.31 ± 1.01
Work place	2.28 ± 0.72
Professional title promotion	2.27 ± 0.87
Opportunity to use and improve professional skills	2.25 ± 0.94
Medicine and medical equipment	2.14 ± 0.91
Long-term engagement in primary healthcare	2.12 ± 0.65
Social status	2.04 ± 0.87
Salary and benefits	1.82 ± 0.83

#### Influencing factors

Chi-square test identified that understanding relevant policies when enrolling in RTME, recognition of rural medical work, relevance of school training provided in RTME with present job, education background, place of origin and salary were significantly correlated with job satisfaction (*p* < 0.05) (Table 4). Whereas age, whether they were the only child, years of service and marital status had no statistical significance on job satisfaction of village doctors.

**Table 4:** Factors influencing job satisfaction of village doctors in the RTME program (*N* = 223).

Variables	Category	Percentage	Overall job satisfaction			Satisfaction rate	*X* ^2^	*t*	*p*
			Dissatisfied	Neutral	Satisfied				
Understanding of relevant policies when enrolling in RTME	Totally unaware	5.83	6	5	2	15.38	16.72	3.223	0.009
	Unaware	21.52	15	17	16	33.33			
	Basically aware	56.95	25	52	50	39.37			
	Fully aware	15.70	2	6	27	77.14		1.956	
Recognition of rural medical doctors	Fully supportive	25.56	3	13	41	71.93	42.241	<0.01
	Partly Supportive	58.30	29	60	41	31.54		
	Not supportive	13.90	14	13	4	12.90		
	Totally unsupportive	2.24	5	0	0	0		
Relevance of school training provided in RTME with present job	Quite relevant	60.54	8	46	81	60	88.074	1.099	<0.01
	Relevant	25.56	8	39	10	17.54			
	Irrelevant	13.90	25	2	4	12.90			
Education Background	3 year Diploma	62.33	45	39	55	39.57	91.371	1.877	<0.01
	Bachelor degree	37.67	47	26	11	13.09			
Place of origin	Rural	92.83	41	52	114	55.34	90.134	1.771	<0.01
	Urban	7.17	10	5	1	6.25			
Salary (CNY/month)	<2,500	2.24	1	3	1	20	17.269	3.002	0.005
	2,600–3,999	32.74	28	20	25	34.25			
	4,000–4,999	52.02	19	56	41	35.34			
	>5,000	13.00	6	12	11	37.93			

Univariate logistic regression analysis was conducted by putting respondents who chose “satisfied” and “neutral” together, and setting “satisfied” and “neutral” as “1,” “dissatisfied” as 0, and then encoding other variables. The logistic regression analysis of factors influencing job satisfaction of village doctors indicated that the influence of the variables on job satisfaction has statistical difference (*p* < 0.05) (Table 5).

**Table 5:** Univariate logistic regression analysis of factors influencing job satisfaction of village doctors in the RTME program (*N* = 223).

Variables	Category	Job Satisfaction		OR	*t*	*P*	95%CI
		Dissatisfied	Satisfied or neutral				
Understanding of relevant policies when enrolling in RTME	Totally unaware	6	7			0.012	
	Unaware	15	33	2.265			0.657–7.806
	Basically aware	25	102	4.191	3.40		1.300–13.516
	Fully aware	2	33	13.417			2.195–81.997
Recognition of rural medical doctors	Fully supportive	3	54	18.222		<0.001	4.724–70.287
	Partly Supportive	29	101	4.578	8.48		2.092–10.019
	Not supportive	14	17				
	Totally unsupportive	5	0				
Relevance of school training provided in RTME with present job	Quite relevant	8	127	31.200		<0.001	10.667–91.258
	Relevant	8	49	26.000	8.034		7.664–88.206
	Irrelevant	25	6				
Education Background	3-year Diploma	45	94	21.342	5.829	<0.001	6.731–68.413
	Bachelor degree	47	37	15.481			3.862–67.532
Place of origin	Rural	41	166	26.531		<0.001	9.693–72.614
	Urban	10	6	12.245	14.029		4.380–34.231
Salary (CNY/month)	<3,999	29	49	0.427		0.002	0.155–1.176
	4,000–4,999	19	97	1.444	3.06		0.510–4.089
	>5,000	6	33				

Multivariate logistic regression analysis was further done to verify the role of each influencing factor on job satisfaction. It has been identified that relevance of school training provided in RTME with present job and place of origin are the independent factors influencing job satisfaction (*p* < 0.01) (Table 6). Compare the results with those of univariate regression, the significance of coefficients of “recognition of rural medical doctors,” “education background” and “salary” reduced to be significant at the level of *p* < 0.1. For the variable “understanding of relevant policies when enrolling in RTME,” it became not significant any more. This might lie in that, the better the “understanding of relevant policies when enrolling in RTME” is, the better recognition of rural medical doctors and the better recognition of relevance of school training provided in RTME with present job. These will lead to better satisfaction.

**Table 6:** Multivariate logistic regression analysis of factors influencing job satisfaction of village doctors in the RTME program (*N* = 223).

Variables	Category	Job satisfaction		OR	*t*	*P*	95%CI
		Dissatisfied	Satisfied or neutral				
Understanding of relevant policies when enrolling in RTME	Totally unaware	6	7			0.955	
	Unaware	15	33	1.888			0.210–16.977
	Basically aware	25	102	1.733	1.368		0.223–13.469
	Fully aware	2	33	1.666			0.125–22.192
Recognition of rural medical doctors	Fully supportive	3	54	6.216		0.082	1.039–36.131
	Partly Supportive	29	101	3.100			0.975–9.852
	Not supportive	14	17		3.645		
	Totally unsupportive	5	0				
Relevance of school training provided in RTME with present job	Quite relevant	8	127			<0.001	
	Relevant	8	49	20.753	5.825		5.454–78.976
	Irrelevant	25	6	25.007			5.563–112.408
Education Background	3 year Diploma	45	94	7.632	2.731	0.071	2.135–40.461
	Bachelor degree	47	37	5.821			1.012–32.876
Place of origin	Rural	41	166	13.780		<0.001	4.077–46.573
	Urban	10	6	10.884	3.513		2.742–43.202
Salary (CNY/month)	<3,999	29	49	0.590		0.080	0.159–2.182
	4,000–4,999	19	97	1.857	2.947		0.512–6.730
	>5,000	6	33				

### Qualitative findings

The following could be summarized through the interviews with 40 interviewees:

#### Expectations on the RTME program

Over 60 percent (25 out of 40) interviewees stated that schools should innovate curriculum design for the RTME training program. More medical courses in the rural context should be incorporated into curriculum, and clinical observations and internship in village hospitals should be arranged from time to time. Courses on professional identity and career planning oriented for rural medical work should be offered.

One interviewee said that:

Most of us attended this program because of lower entry requirement and free tuition. As far as I know, most of us have low professional identity, and do not want to be a village doctor. We have ever regretted or complained when we study in college or work in the village hospitals. You see, in college, we were grouped in a separate class, but we had exactly the same courses as other medical students. We had signed the contract and all of us had to work in the rural hospitals after graduation. The school should offer tailor-made courses focusing on rural settings to help us better adapt to the work in village hospitals, especially career planning courses and specialty courses.

Seventy-five percent (30 out of 40) interviewees expected the RTME program to offer more preferential policies, especially in professional title assessment and further education, to doctors who have fulfilled the contract to work in village hospitals as stipulated.

### Expectations on the job

Over half of the interviewees (22 out of 40) said that they had such heavy workloads that they often felt exhausted and had no time to receive on-the-job training or do scientific research. In addition to basic healthcare to rural residents, they were required to undertake 11 public healthcare service, such as setting up health care records, health education, report and treatment of infectious diseases, and chronic disease prevention control.

All the 40 interviewees expected that the salary and benefits could be further improved, and housing fund could be higher.

Inadequacy of basic medicine and medical equipment was a common problem, which affected the diagnosis and treatment of diseases and hindered the improvement of medical skills.

One male village doctor said that:

I had met a Type II diabetic patient whose blood sugar could not be controlled well by oral medication, and insulin was highly recommended. But we had no insulin here, so, I couldn’t help the patient. Then, the patient had to go to municipal hospital. It made me feel frustrated.

### Suggestions to future medical students in the RTME program

Approximately 78% (31 out of 40) of the interviewees stated that they would recommend others to join in the RTME program because of lower entry requirement, free tuition, and guaranteed employment. Graduates of the RTME program either have junior college diploma or a bachelor’s degree in clinical medicine. Generally speaking, it is hard for students without master’s degree to land a job as a doctor in hospitals in China. But this program provides a healthcare position for them.

Before enrolling, it is suggested to know more about the RTME policies, village doctors’ duty and rural hospitals. While on the RTME training program in colleges, it is advisable for students to form a positive professional identity, make career plans and have a deeper understanding of future work as primary healthcare providers. In this way, they can better adapt to the job in village hospitals. More attention should also be paid to the improvement of interpersonal skills, learning how to communicate with rural residents effectively.

One interviewee said that:

I only got 348 (total score 750) in the entrance exam for colleges, and could only be admitted to a three-year vocational school. I have dreamed to be a doctor, but it is almost impossible to work as a doctor without a master’s degree in this competitive labor market. With the RTME program, I can study clinical medicine free of charge and become a doctor with a diploma only. So, I do think it is a fairly good chance.

### Reasons to continue working in village hospitals when the contract expires

According to the respondents, continuous supports and preferential policies are the fundamental driving forces for them to continue working in village hospitals when the contract expires. Less competition and more promotion opportunity are important extrinsic factors. Sense of achievement is a primary intrinsic factor for the graduates of the RTME program to remain in rural areas. Salary is not considered to be closely relevant.

The 2021 Statistical Yearbook issued by the National Health and Family Planning Commission of the People’s Republic of China reported that, in 2020, 0.3% of village doctors had a bachelor’s degree and 5.4% had a junior college diploma. Therefore, graduates of the RTME program are often thought highly by local hospitals and given more chances and attention, which in turn, contributes to the willingness to remain in village hospitals.

One doctor who has worked for 4 years said that:

Since I studied in the medical college, our teachers have introduced relevant preferential policies towards rural health care and general practitioners by the Chinese government. When I started working here, I have witnessed the great changes in village hospitals. More attention and chances have been given to us. So, I think support from the government will be one of the most important reasons for me to continue working here. I believe there will be better policies for village doctors in the future.

Another doctor described:

I love the atmosphere here a lot. In our hospital, we do not need to do research and write papers in order to get promoted in technical title. So we just need to focus on treating patients and improving clinical skills. This makes me feel good. My classmates who are working in the city are extremely stressful since there is fierce competition in technical title assessment. They have to work hard both in clinics and research. Therefore, I should say the relaxing atmosphere and favorable policy count a lot [sic].

A newly-graduated doctor stated that:

I am not sure whether I will stay here when the contract expires. But I can see the government is laying more emphasis on village hospitals. The working conditions are not quite good, but things are getting better. Actually, I like working here because of strong sense of achievement by helping local residents relieve pain with limited medicine and medical equipment. What’s more, there is lower requirement in professional title assessment for us, such as years of service, research projects or papers. Maybe right now the salary is not very high, but as we work longer and have higher professional title, we can earn more. So, considering sense of achievement, professional title promotion and development potential, most probably I will stay here.

## Discussion

This study sought to provide insights into job satisfaction of village doctors in the Rural-oriented Tuition-waived Medical Education (RTME) program in China. Our findings highlighted four themes:

### Village doctors with a rural origin have higher job satisfaction

Male village doctors with a rural origin reported higher job satisfaction in this study. A great deal of evidence has suggested that health workers with a rural background are more willing to work in rural areas (Serneels et al., 2010). Similarly, medical students with rural origins are more willing to work in rural areas compared with those with an urban background (Deressa and Azazh, 2012; Sapkota and Amatya, 2015).

### Full understanding of the RTME program is the precondition of job satisfaction

This study reported that better understanding of the RTME was correlated with higher job satisfaction. Those who were fully aware of relevant policies of the RTME program only accounted for 15. 70% (35/223) were not aware, which indicated a relatively poor understanding and acceptance of the RTME program.

Previous studies have identified that intrinsic motivation, i.e., the desire to do something for its own sake (Xiong et al., 2016), can have a strong effect on job satisfaction. However, the participants in this study said they were mainly attracted by extrinsic factors, such as the benefits of guaranteed employment and free tuition, and did not fully understand the very nature of rural medical work during enrollment, which is in accordance with findings in previous studies (Wang and Hu, 2011; Ren et al., 2018).

It is suggested that relevant authorities should strengthen publicity through various channels, explaining and interpreting RTME policies, training scheme, primary healthcare, rural medical work, payment and possible career paths in details. Then the students can make more rational decisions to choose the RTME program due to enthusiasm about primary health care, which may contribute to higher job satisfaction and retention rate.

### Renumeration is a critical factor of job satisfaction but not an important indicator of retention in village hospitals

Prior studies (Chan et al., 2005; Zhang and Fang, 2016; Budhathoki et al., 2017; Hu et al., 2018; Song and Mei, 2018; Mohammadiaghdam et al., 2020; Wang et al., 2020) reported that low salary was negatively correlated with low job satisfaction of village doctors. This study verified this finding. However, the semi-structured interviews indicated salary was not directly correlated with future retention in village hospitals.

To improve job satisfaction, measures to increase the retention of the village doctors with RTME rather than simply recruiting them should be given greater attention (Meng et al., 2009), for instance, by providing appropriate and adequate local infrastructure and setting competitive remuneration in rural medical institutions (Buykx et al., 2009; Agyei-Baffour et al., 2011; Song and Mei, 2018; Wang et al., 2020). *Incentive Mechanism for the Training and Use of General Practitioners*, issued by the State Council of China in 2018, has mandated competitive remuneration and salary reform. But it takes time to fully implement these policies nationwide.

### Optimizing curriculum design of RTME training is pressing

As indicated in the investigation, the relevance of school training provided in RTME with present job was positively correlated with job satisfaction. The semi-structured interviews revealed that there was a lack of career planning and clinical courses specially designed for rural settings, and inadequacy of observations and internship in rural hospitals, which made these medical students in the RTME program have low professional identity and find it hard to adapt to the job in the very beginning. Improving the educational scheme in the medical college is conducive to the students’ job satisfaction and intention of remaining in rural areas when the contract expires.

Efforts should be made to build on intrinsic motivation (i.e., professional identity) during medical training (Agyei-Baffour et al., 2011). College and internship are critical periods when professional identity is formed and the medical colleges are supposed to provide individualized career planning courses, which is vital to the formation of professional identity and future job satisfaction (Chen et al., 2020).

Previous research identified that the curriculum design for RTME program was not reasonable and practical. Most colleges put students in the RTME program in one class, having lessons separately. But there were no tailored courses for them. One to 6 months’ internship was arranged in community hospitals instead of village hospitals. So, many graduates found it hard to adapt when they started working in the countryside (Zhang, 2018). Related studies have suggested improving the curricular design (i.e., matching curricula with rural health needs and implementing early exposure and experience to rural medical work, introduction of Community-Based Education and Service or similar curriculum components) and early exposure to rural settings could increase medical students’ interest in rural medical practice (Buchan et al., 2013; Qing et al., 2015; Amalba et al., 2018; Ishimaru et al., 2019).

## Conclusion

Through investigations into village doctors in the Rural-oriented Tuition-waived Medical Education (RTME) program in China, the pivotal members in primary health care teams, it was revealed that 56.50% (126/223) of the village doctors in the RTME program were satisfied with rural medical work. Doctors with good understanding of RTME policies and high recognition of rural medical work had the highest satisfaction; doctors whose school training provided in RTME was relevant to present job also had a higher level of satisfaction; similarly, doctors with rural origins and high salary reported a higher level of satisfaction. Relevance of school training provided in RTME with present job and place of origin were the independent influencing factors.

Based on in-depth interviews, preferential policies of RTME program, relaxing working atmosphere, more promotion opportunity, and easier access to higher technical title were the key factors for the graduates with RTME program to remain in rural areas. Renumeration was not considered to be closely relevant in terms of retention.

### Implications

This study has a number of implications. First, the RTME program should attract and enroll more medical students with rural background due to the significant association between job satisfaction in village hospitals and rural origin. Second, RTME training should be optimized by providing tailor-made curriculum relevant to primary healthcare, offering individualized career planning courses for village doctors and arranging longer exposures to village hospitals during internships, in order to cultivate the ability to perform rural medical work, improve the cognition of the value of rural medical work and elevate professional identity of village doctors. Third, authorities are expected to offer supportive mentorship system and preferential policies, especially in terms of professional title assessment and continuing education.

### Limitations and future research

Since the RTME program was initiated nationwide from 2010, the number of graduates was not large enough. This study focused on village doctors with RTME program in Anhui, China only, so the samples were relatively small and limited. In addition, telemedicine counseling was used during the COVID-19 pandemic, which might have an effect on job satisfaction. The questionnaire in this study, a well-received measurement tool that has been used in prior studies in China, did not include this factor. We expect that future research can fill in these gaps. It is recommended that more research should be done to a broader range of primary health care providers. Additional studies among rural doctors of this particular group can be conducted with a larger sample size. Second, a longitudinal study is highly recommended to analyze the causal effects of birth place, understanding of RTME policy, salary and curriculum design of RTME training on job satisfaction. The interactive effects between explanatory variables should be further deeply explored. Third, the need for tailored courses for rural doctors has been advocated, therefore institutions can develop curriculum and mentorship program accordingly, which is an open area for further research.

## Data availability statement

The raw data supporting the conclusions of this article will be made available by the authors, without undue reservation.

## Ethics statement

The studies involving human participants were reviewed and approved by health commission of Anhui Medical College (Decision no: 2021. 09. 30). The patients/participants provided their written informed consent to participate in this study.

## Author contributions

YC designed, conducted the research, and wrote the paper. RJ analyzed the data and did the proofreading. All authors contributed to the article and approved the submitted version.

## Funding

The authors disclosed receipt of the following financial supports for the research, authorship, and/or publication of this article: This work was supported by Anhui Ministry of Education [grant number SK2021A0884], the National Office for Philosophy and Social Sciences of China [grant number 21BGL072], Anhui Social Science Foundation [grant number AHSKXZX2020D01], Anhui Ministry of Education [grant number 2020mooc243].

## Conflict of interest

The authors declare that the research was conducted in the absence of any commercial or financial relationships that could be construed as a potential conflict of interest.

## Publisher’s note

All claims expressed in this article are solely those of the authors and do not necessarily represent those of their affiliated organizations, or those of the publisher, the editors and the reviewers. Any product that may be evaluated in this article, or claim that may be made by its manufacturer, is not guaranteed or endorsed by the publisher.
